# Comparison of Genomic Prediction Methods for Yellow, Stem, and Leaf Rust Resistance in Wheat Landraces from Afghanistan

**DOI:** 10.3390/plants10030558

**Published:** 2021-03-16

**Authors:** Muhammad Massub Tehseen, Zakaria Kehel, Carolina P. Sansaloni, Marta da Silva Lopes, Ahmed Amri, Ezgi Kurtulus, Kumarse Nazari

**Affiliations:** 1 Department of Field Crops, Ege University, P.O. Box 35100 Bornova, Izmir, Turkey; massub.tehseen@gmail.com; 2 International Center for Agricultural Research in the Dry Areas (ICARDA), ICARDA-PreBreeding & Genebank Operations, Biodiversity and Crop Improvement Program, P.O. Box 10000 Rabat, Morocco; Z.Kehel@cgiar.org (Z.K.); A.amri@cgiar.org (A.A.); 3 International Maize and Wheat Improvement Center (CIMMYT), Carretera México-Veracruz Km. 45, El Batán, Texcoco C.P. 56237, Mexico; C.Sansaloni@cgiar.org; 4 Sustainable Field Crops Programme, IRTA (Institute for Food and Agricultural Research and Technology), 25198 Lleida, Spain; marta.dasilva@irta.cat; 5 International Center for Agricultural Research in the Dry Areas (ICARDA), Biodiversity and Crop Improvement Program, Regional Cereal Rust Research Center (RCRRC), P.O. Box 35661 Menemen, Izmir, Turkey; E.Kurtulus@cgiar.org

**Keywords:** genomic prediction, wheat landraces, yellow rust, leaf rust, stem rust

## Abstract

Wheat rust diseases, including yellow rust (Yr; also known as stripe rust) caused by *Puccinia striiformis* Westend. f. sp. *tritici*, leaf rust (Lr) caused by *Puccinia triticina* Eriks. and stem rust (Sr) caused by *Puccinia graminis* Pres f. sp. *tritici* are major threats to wheat production all around the globe. Durable resistance to wheat rust diseases can be achieved through genomic-assisted prediction of resistant accessions to increase genetic gain per unit time. Genomic prediction (GP) is a promising technology that uses genomic markers to estimate genomic-assisted breeding values (GBEVs) for selecting resistant plant genotypes and accumulating favorable alleles for adult plant resistance (APR) to wheat rust diseases. To evaluate GP we compared the predictive ability of nine different parametric, semi-parametric and Bayesian models including Genomic Unbiased Linear Prediction (GBLUP), Ridge Regression (RR), Least Absolute Shrinkage and Selection Operator (LASSO), Elastic Net (EN), Bayesian Ridge Regression (BRR), Bayesian A (BA), Bayesian B (BB), Bayesian C (BC) and Reproducing Kernel Hilbert Spacing model (RKHS) to estimate GEBV’s for APR to yellow, leaf and stem rust of wheat in a panel of 363 bread wheat landraces of Afghanistan origin. Based on five-fold cross validation the mean predictive abilities were 0.33, 0.30, 0.38, and 0.33 for Yr (2016), Yr (2017), Lr, and Sr, respectively. No single model outperformed the rest of the models for all traits. LASSO and EN showed the lowest predictive ability in four of the five traits. GBLUP and RR gave similar predictive abilities, whereas Bayesian models were not significantly different from each other as well. We also investigated the effect of the number of genotypes and the markers used in the analysis on the predictive ability of the GP model. The predictive ability was highest with 1000 markers and there was a linear trend in the predictive ability and the size of the training population. The results of the study are encouraging, confirming the feasibility of GP to be effectively applied in breeding programs for resistance to all three wheat rust diseases.

## 1. Introduction

Wheat is one of the most important cereal crops of the world, grown from the Equator to the Arctic Circle, and considered a staple food of 35% of the world population, which serves as the major source of carbohydrates in the human diet [1,2]. The three rust diseases of wheat, namely yellow rust (Yr), stem rust (Sr), and leaf rust (Lr), are a continuous threat to wheat production around the world [3]. Several genes for resistance to each of the three rusts have been catalogued and genetically characterized [4], however, many of the known genes have now become ineffective, that is susceptible, to newly virulent races of the pathotypes. Rusts have been detected in areas where they had not been detected before. Therefore, a continuous search for new sources of resistance is of paramount importance in the fight against the wheat rust diseases [5]. Fungicides have been used to control wheat rusts; however new races frequently develop resistance to commonly used fungicides, complicating the control of these diseases [6]. Moreover, environmental concerns have prompted some countries (e.g., the EU) to impose regulations to restrict the amounts of chemical products used in agriculture (Directive 2009/128/EC), also reinforcing the importance of ensuring rusts resistance in modern varieties.

Wheat landraces are an important potential source of diversity for breeding and pre-breeding germplasm, primarily due to the co-evolution with biotic and abiotic stresses throughout history [7]. These landraces often contain untapped resistance for many biotic and abiotic stresses, such as for example the wheat rusts [8]. In a recent study, it was concluded that genetic enhancement programs can be directly initiated from landraces [9]. There are two types of genetic resistance against the wheat rusts: (i) seedling (qualitative, vertical, and/or all stage resistance) which is usually effective throughout the life of the plant. However, it is controlled by race-specific genes and may not provide broad-spectrum resistance to all races unless the needed genes are pyramided into single plant genotypes and (ii) APR (quantitative resistance, partial resistance or horizontal resistance) which is expressed as the plant reaches its reproductive stage [3], and most likely provides protection to all races at varying levels. APR is usually non-race-specific, but it can also be race-specific in the case of *Yr11, Yr12, Yr13,* and *Yr14* [10]. Either way, APR is considered more durable over time, providing long-term resistance, than race-specific, seedling resistance [11]. The precise phenotyping for APR in field conditions is an expensive and labor-intensive undertaking. The average cost of the most basic field assay in CIMMYT field experiments was estimated to be around US$30 to 40 per accession planted in two replications in a single location [12]. This cost is increased many-fold when experiments are conducted in glasshouses while the current molecular-genotyping cost of a single genotype is less than US$20 [13]. Genomic prediction (GP) is a technique that can help increase the rate of genetic gain per unit price and reduce the length of breeding cycles [14]. Genomic prediction is carried out in two steps, the genotypic marker information of genotypes is used to predict the genomic estimated breeding values (GEBVs), and selection of new germplasm is developed via hybridization based on these GEBVs before field experiments [14]. Thus the number of genotypes and unit cost of the experiment is reduced, thereby increasing genetic gains [15]. Therefore, GP studies are primarily focused on developing methods and models to estimate GEBV with as much accuracy as possible. In wheat, GP studies have been reported in a diverse set of populations from global wheat lines in CIMMYT [16] to bi-parental [17] and, multi-parental [18] populations, and landrace collections [8,19,20]. The success of highly accurate estimations of GEBVs and model calibration depends upon intensive phenotyping and genotyping of the training population, which is then used to estimate the GEBV of the genotypes in the actual test set. The type and number of genotypes in the training population affects the prediction accuracies of the different models used for GP [14]. Several studies have shown the potential of GP for various quantitative traits including resistance to rust diseases in wheat [8,12,14,15,19,21]. Many GP models have been developed and tested before [14]. In general, the models differ according to how the marker effects are treated and the way the models account for population structure. There are several parametric, semi-parametric, and nonparametric models for GP. The Genomic Best Linear Unbiased Prediction (GBLUP) and Ridge regression (RR) models treat all marker effects as the same, whereas the Bayesian models treat markers with different effect sizes differently.

This study aims to compare and evaluate the GP accuracies of nine different methods including parametric, semi-parametric, and Bayesian models to predict APR for yellow, leaf, and stem rust of wheat in a panel of bread wheat landraces from Afghanistan. We also investigated whether the number of landraces in the training population and the numbers of markers used in the analyses have an effect on the predictive ability of the model used for GP.

## 2. Results

### 2.1. Phenotypic Data and Heritability

**Figure 1:**
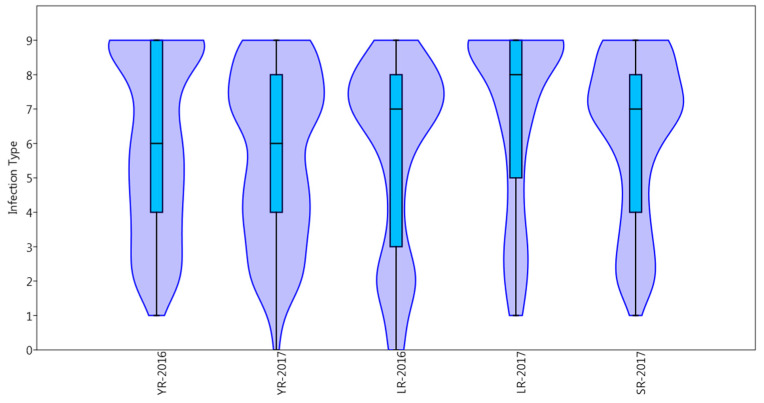
Violin and box plots of phenotypic distribution for yellow (YR), leaf (LR) and stem (SR) rusts, in 2016 and 2017, under field conditions.

The phenotypic distributions for all traits are shown as percentages in Table 1, and as violin and box plots in Figure 1. The landraces showed the highest percentage of resistant reaction to leaf rust in 2016 (21.5%), while the lowest percentage of resistant landraces was observed in leaf rust in 2017 (9%). In the case of yellow rust the landraces showed similar reaction types in both years. The Pearson correlation coefficient for yellow rust across the two years was high (0.70). The mean correlation between leaf rust years 2016 and 2017 was moderate (0.4). The correlation between stem and leaf rust was also moderate (0.5). There was no significant correlation between yellow rust and the other leaf and stem rust scores (−0.2 and −0.1) (Table 2).

### 2.2. Marker Distribution and Genetic Relationship Matrix

**Figure 2:**
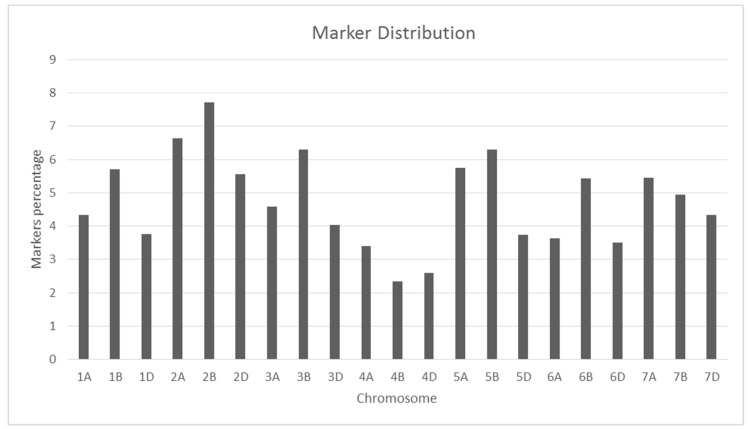
Percentage of markers used in the study on 21 chromosomes of wheat.

**Figure 3:**
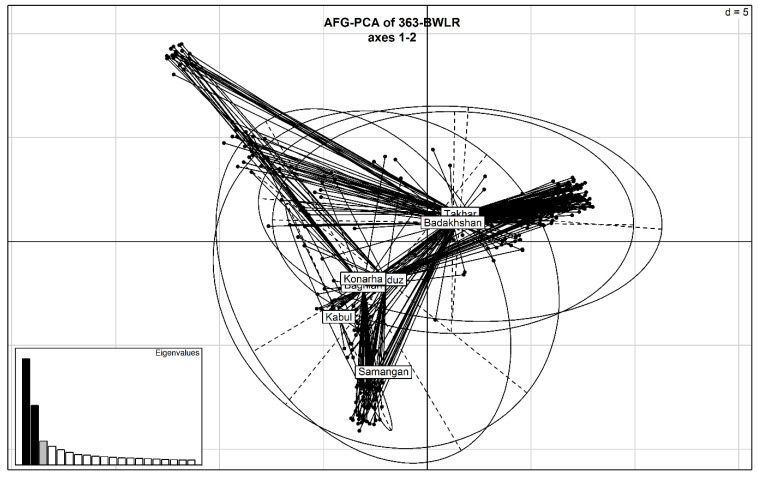
Principal Component Analysis of 363 Afghan bread wheat landraces.

From a total of 24K markers, a set of 11,428 markers was used in this study after filtering the markers on missing information and minor allele frequency. The markers with known genetic positions were distributed across the whole A, B, and D genomes (Supplementary Table S1). The percentage of markers on the A, B, and D genomes was 33.4, 38.3, and 27.2%, respectively. The highest number of markers were distributed on chromosomes 2B and 2A, whereas chromosomes 4D and 4A had the lowest number of markers (Figure 2). There were three clusters in the Principal Component Analysis among the 363 bread wheat landraces collected from seven geographic regions in Afghanistan. The first three principal components explained 19.6% of the genetic variation. However, there was no clear clustering by the geographic origin of the landraces (Figure 3). The genomic relationship matrix showed a high degree of relatedness amongst the landraces, making them suitable for genomic prediction analysis (Supplementary Figure S1).

### 2.3. Genomic Predictive Abilities of the Nine Methods for Yellow Rust

Table 3 shows that for the yellow rust evaluation trial in 2016, the model that gave the highest prediction accuracy (0.33) was RKHS. While LASSO and EN both gave the lowest prediction accuracies, they did not deviate significantly from the other models. Overall, the accuracies for the GBLUP, RR, BRR, BA, BB, and BC models for yellow rust during 2016 were similar. The prediction accuracies for yellow rust in 2017 followed the same pattern as in 2016 except that the RKHS model was not the best in predicting yellow rust as was the case in 2016. LASSO and EN gave the lowest accuracies for yellow rust also in 2017. The prediction accuracies for GBLUP, RR, BRRM, BA, BB, and BC were similar.

### 2.4. Predictive Abilities of the Different Methods for Leaf Rust

For leaf rust in 2016, the GBLUP, RR, BRR, and BB models gave the highest prediction accuracies (Table 3). The two models LASSO and EN gave the lowest prediction accuracies but not much significantly different from the others. The accuracy for RKHS was slightly better than LASSO and EN. For the leaf rust in 2017, none of the models was able to predict the landraces’ GEBV scores. This may be because of too many missing observations in the training subsets, which did not allow the models to predict the landraces. Stem rust predictive abilities of the different methods. For the stem rust dataset, the models that gave the highest prediction accuracies were LASSO and EN, followed by BRR and BB (Table 3). The RKHS model gave the lowest prediction accuracy for stem rust disease. The prediction accuracies from GBLUP, RR, BA, and BC models were similar. LASSO and EN model gave the highest prediction accuracy only for the stem rust dataset whereas in the case of the yellow and leaf rust datasets they were the least performing models.

### 2.5. Comparison between the Models

**Figure 4:**
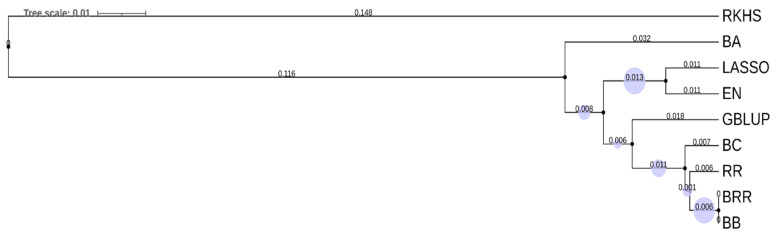
Unweighted Pair Group Method with Arithmetic Mean cluster dendrogram based on the predictive abilities of the different genomic prediction methods.

Based on the predictive abilities for all the models, it was clear that none of the single models outperformed the rest for each of the three rusts and in both years. Overall, the GBLUP, RR, BRR and BB models gave the highest prediction accuracies than other models for most traits. The models LASSO and EN gave the lowest prediction accuracies for yellow and leaf rust, and the highest prediction for stem rust. A cluster dendrogram showing the hierarchical clustering of the prediction models separates the LASSO and EN models from the rest of the models. Also the regression models were far from the Bayesian models in Figure 4. The Spearman rank correlation coefficients between the GEBVs between all models (Table 4) show that all the models were highly correlated to each other. The LASSO and EN models had the lowest correlation coefficient (0.82) with the GEBVs of the other models. The correlations between the GEBVs among the other models were unity or close to unity.

### 2.6. Simulation Analysis

**Figure 5:**
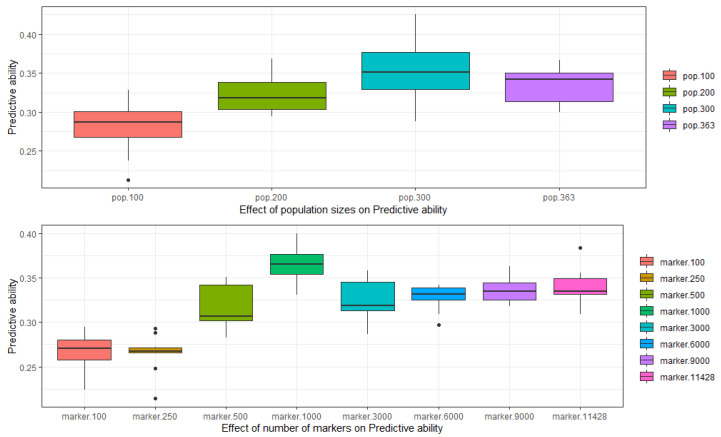
Effect of different population sizes and the number of markers on the predictive ability of genomic prediction.

We ran two different simulations to see the effect of training population size and the number of markers on the prediction accuracy. The GBLUP method was used to compare the effect on prediction accuracy. The whole panel of 363 bread wheat landraces was divided into four subsets of 100, 200, 300, and a whole set of 363 landraces. The prediction accuracy was lowest in the subset of training population with 100 landraces with an increasing trend as the population size increased. In the second simulation, the genotypic data was divided into a randomly selected set of markers from 100, 250, 500, 1000, 3000, 6000, and 9000 to the full set of 11,428 markers. The prediction accuracy was lowest with the set of 100 and 250 markers and as the numbers of markers increased so the prediction accuracy till the set of 1000 markers where the prediction was highest beyond that, no significant progress was observed (Figure 5).

## 3. Discussion

Globally the wheat cereal rusts are the economically most devastating diseases of wheat crops [22]. Previously many studies have predicted important wheat diseases including rusts with different genomic prediction models using elite lines, cultivars and landraces [8,15,19,20,23]. Here we investigated the prediction accuracy of nine genomic prediction methods to predict wheat rust disease responses in a panel of landraces of Afghanistan origin preserved in ICARDA’s gene bank. The genomic relationship of training and testing populations has been reported to be an important factor in predicting GEBVs with high accuracy, and is therefore of paramount importance in the development of training and testing populations [24].

There was no clear clustering among the landraces with respect to their origin. However, a high degree of relatedness among the landraces made them a good population for genomic prediction analysis.

There was no significant correlation between the reaction to yellow rust and the reaction to the other leaf and stem rust, indicating possibly different genetic resistance bases between them.

Among the wheat rusts evaluated in the study, leaf rust (0.38) had the highest mean genomic prediction accuracies followed by both yellow rust from the 2016 dataset and stem rust (0.33) and the least prediction accuracy was observed in yellow rust dataset from 2017 (0.30). The genomic prediction accuracies were moderate with average prediction accuracy of 0.32 across all rusts. The level of prediction accuracy is similar to previously published work on maize and wheat [16,18] The results were consistent with a previous study of genomic prediction analyses for all three wheat rusts in landraces using GBLUP and Bayesian Regression or BRR [8]. The nine models used in the study gave almost similar prediction accuracies. However, the prediction accuracies from LASSO and EN methods were the lowest for yellow rust and leaf rust, but the highest for stem rust. Similar trends were obtained in previous studies with no significant differences amongst the different genomic prediction models [20,25]. The GBLUP and Bayesian models investigated in the study gave almost similar prediction accuracies despite considering all marker effects having similar variances in the GBLUP model. Therefore, the use of prior densities for marker effects in all Bayesian models did not yield significantly better prediction accuracies. Hence, both models can be used for prediction of complex traits like wheat rusts. However, since we did not observe any significant differences in regression-based GBLUP and RR methods, and the Bayesian-based models, the assumption of marker effects having equal variances proved to be effective for the rust traits that were analyzed in this study. Thus, the higher computational time required for prior densities and shrinkage of the Bayesian models may not be needed.

Many previous studies have reported similar prediction accuracies for GBLUP and Bayesian methods in different populations and for different traits [12,15]. The models BA and RR gave similar prediction accuracies in genomic prediction studies in dairy bulls [26]; BC and RR models gave same prediction accuracies in oats for yield, heading and plant height [27]; BA, BB, and BC methods had similar prediction accuracies as RR in North American Holstein bulls [28]; and in a study of *Fusarium* head blight the RR, BC, and BL models gave similar prediction accuracies [13]. The GBLUP or RR models are also reported to give similar prediction accuracies as the BC and BL methods in stem and yellow rust of wheat [12,21]. In some studies Bayesian models have given slightly better prediction accuracies than GBLUP and RR methods: BC and BA gave better accuracy over RR-BLUP in a study of different quantitative traits in Loblolly pine (*Pinus taeda* L.) [29]; and BA and BB excelled over GBLUP in simulation studies [24,30]. However, other studies reported higher predictive ability of GBLUP over its Bayesian counterparts, especially with traits controlled by large-effect QTL [31,32].

The nonparametric models such as RKHS have been reported to show better prediction accuracy than parametric models like GBLUP and RR [16,33]. In our study RKHS gave somewhat similar predictive accuracies as GBLUP and the results are consistent with studies concluding that there was no clear advantage of one model over the other [34]. However, the Bayesian models performed better than RKHS in leaf and stem rust GEBV prediction. LASSO and EN models predicted the lowest prediction accuracies for yellow rust and leaf rust while highest for stem rust, both the models predict GEBV’s by assuming that some markers have large effects on the trait in study and thus may suggest the presence of casual genes with large effect in case of stem rust and it needs to be further investigated.

The choice of the best genomic prediction model is the key to successful GEBV predictions [25]. The accuracy of prediction ability of a model depends upon several factors including trait heritability, marker coverage and density, and the size of the training population. The relatedness between training and validation population also aids in selecting the most accurate model for prediction of traits under study in specific populations [24,31,33,35]. All the genomic prediction models gave almost zero GEBV outcomes for the leaf rust 2017 dataset. This may be attributed to a lot of missing observations in the data and as a result, the training population was too small to be able to predict the GEBVs.

To investigate the effect of population size on prediction accuracy we divided the whole population into four subsets and compared the accuracies in all the subsets using the GBLUP method. The results indicated that the increase in the number of genotypes included in the training populations resulted in an increase in prediction accuracy until a certain level (*n* = 300), being consistent with previous studies [25]. The choice of the prediction methods becomes more important with an increase in the number of training populations [36].

Similarly, to investigate the effect of the number of markers on the prediction ability of a model the marker data was divided into eight subsets. As reported in previous studies, the GBLUP model gave better and higher accuracies with an increasing number of markers [31,37]. The prediction accuracy from using 500 markers is better than 100 and 250 markers and 1000 markers predicted better GEBV’s than 500 markers but after 1000 markers not much gain could be made. Studies have reported that genomic prediction accuracy is not influenced after a certain number of markers is implemented [38]. Further investigations with increasing marker density could provide more clear insights, but in previous reports it was observed that even analysis with combinations of dense 35k and 90k markers did not increase prediction accuracy [23]. Therefore, we concluded that the whole panel of 363 landraces with 1000 markers can give roughly the same prediction accuracy all 11k markers.

The hierarchal clustering of the nine genomic prediction models using the GEBVs grouped them as expected, where regression models grouped together and Bayesian models were clustered together. The RKHS model was placed far from both regression and Bayesian models. LASSO and EN gave the lowest prediction abilities for Yellow rust and Leaf rust whereas highest for Stem rust and were as expected grouped together in the cluster analysis. The clustering of the models based on the predicted GEBVs were similar to a previous study [15].

Our results reveal the potential use of landraces from Afghanistan as valuable genetic resources for breeding programs focused on rust resistance in wheat. The GP accuracies obtained in the studies were moderate and consistent with previous studies of genomic prediction for rust diseases [8,12,21], and this magnitude of genomic prediction is considered useful for the prediction of other landraces preserved in gene banks [19]. The landraces preserved in the gene banks can be improved using conversion (improve landraces until they become elite material) and introgression (landraces crossed with elite material) approaches. Genomic prediction has been proven to be a useful strategy in improving genetic merit with either strategy [9]. The current study shows the potential of genomic prediction for enhancing breeding for rust resistance in wheat and concludes that models like GBLUP with light computational capability can predict the GEBV similarly well compared to other models which require more computational time. Moreover, for GP studies the number of markers can be optimized to save computational time and that the efficacy of accurate GP can be increased with an increase in the size of the training population. The GP models can be utilized to predict the GEBVs of other landraces preserved in the gene banks and after verification of GEBVs with phenotyping in natural conditions, outstanding parental stocks can be selected for future crossing with either of the strategies discussed above to accelerate genetic gain over time.

## 4. Materials and Methods

A panel of 363 bread wheat landraces conserved in the ICARDA gene bank and collected from seven geographic regions of Afghanistan was used in the study. The highest number of landraces were collected from Badakhshan and Takhar regions with 119 accessions from each region. The rest of the accessions were collected from Baghlan (*n* = 66), Kabul (*n* = 7), Konarha (*n* = 1), Kunduz (*n* = 50), and Samangan (*n* = 1) (Supplementary Table S2). The panel was screened for adult plant resistance (APR) against yellow rust, leaf rust, and stem rust.

### 4.1. Adult Plant Evaluation and Phenotypic Data

The field experiments were laid out as an augmented design with un-replicated landraces and repeated check rows in 22 blocks. Each block contained 17 landraces and two checks. Thirty seeds from each accessions were planted in a 1-meter rows with 30 cm spacing between the rows. To ensure sufficient inoculum production for disease infection, a mixture of the universally susceptible varieties ‘Morocco’, ‘Seri 82’, and ‘Avocet S’ along with the locally susceptible varieties ‘Bolani’, ‘Basribey’ (from the CIMMYT cross ‘Kauz’), and ‘Cumhuriyet 75’, ‘Kunduru’, ‘Kasifbey’, and ‘Gonen’ was planted as spreader after every 20 rows, as well as spreader rows bordering the nurseries. The experiments were managed as per the standard local agronomic practices during the crop season.

#### 4.1.1. Adult Plant Resistance for Yellow Rust

APR to yellow rust was evaluated under field conditions against the *PstS2* and *PstS7* (Warrior race) races of yellow rust, which had been collected in previous years and preserved at RCRRC (for virulence/ avirulence formula of these two races see Supplemental Table S2) The landraces were inoculated artificially with a mixture of the two races in talcum powder using a backpack sprayer at the seedling stage. The inoculation was repeated twice, at the tillering and booting stage. The field was irrigated with an artificial misting system. The plants were scored when the disease severity reached 100% on the susceptible check Morocco, using the Modified Cobb Scale [39]. The landraces were also recorded for major infection types R, MR, MS, and S, based on the 0–9 scale where disease scores of 0, 1, and 2 were considered resistant 3 and 4 moderately resistant 5 and 6 moderately susceptible and 7, 8, and 9 as susceptible [40].

#### 4.1.2. Adult Plant Resistance for Leaf Rust

The bread wheat landraces were evaluated for APR against leaf rust during the field cropping seasons 2016 and 2017. A leaf rust isolate collected from field infection in 2016 was used in seedling assessment of leaf rust isogenic lines. The virulence/ avirulence formula of this isolate is presented in Supplemental files (Supplementary Table S2). Collected spores in 2016 was used in field inoculations in 2017. Collected spores were suspended in light mineral oil (Soltrol 170) and artificially sprayed twice using an atomizer during the tillering and booting stages. The data was recorded based on disease infection types on the above indicated 0-9 scale. During the 2017 cropping season, due to the high yellow rust epidemic, it became very difficult to distinguish both yellow rust and leaf rust, and therefore only 221 landraces could be recorded that year for leaf rust APR which survived during the leaf rust scoring because of their full susceptibility to yellow rust.

#### 4.1.3. Adult Plant Resistance to Stem Rust

APR to stem rust was evaluated for the bread wheat landraces against naturally occurring local stem rust races during the 2017 cropping season. In race typing of collected samples from the same field using the 20 north American differential lines [41], presence of stem rust race TKTTF was confirmed (Supplementary Table S2). The stem rust response was evaluated based on the above indicated 0–9 scale.

### 4.2. Correlation, Heritability, and Relationship Matrix

The Pearson correlations between the yellow, stem, and leaf rust were performed using XLSTAT 2017 software (Addinsoft, New York, NY, USA) to estimate the relationship between the phenotypic traits under study. Genetic and residual variances and broad-sense heritability were estimated for the trials using the software PBTools (Version 1.4, http://bbi.irri.org/products (accessed on 4 March 2021)). Principal Component Analysis was performed in R software using the pcadapt package [42]. The unweighted Pair group Method with Arithmetic Mean cluster dendrogram was completed using the software PAST (Version 3.0, http://palaeo-electronica.org/2001_1/past/issue1_01.htm (accessed on 4 March 2021)).

### 4.3. Genotyping

The landraces were genotyped using DArT technology using genotyping by sequencing (GBS) method [43] at the Genetic Analysis Service for Agriculture (SAGA) at the International Maize and Wheat Improvement Center (CIMMYT) in Mexico and supported by the Seed of Discovery Project, SeeD. Markers were filtered based on missing data >20%, minor allele frequency (MAF) <5%, and other parameters such as call rate, polymorphic information content (PIC), and reproducibility, that resulted in 11,428 markers. The markers were subjected to imputation before running the genomic prediction model using the EM (Expectation-Maximization) algorithm as implemented in the BWGS package in R [44].

### 4.4. Genomic Prediction Methods

#### 4.4.1. GBLUP and RR-BLUP

Genomic Best Linear Unbiased Prediction (GBLUP) uses a marker-based relationship matrix to predict breeding values [45,46]. GBLUP is a parametric method that uses the additive effect of the markers for the estimation of breeding values. It is strictly equivalent to RR-BLUP (Ridge Regression) in theory. The mixed model used in GBLUP to predict phenotypes of landraces is:

$$y=1_{n}\mu +Zu+\varepsilon$$

where *y* is the phenotypic trait response, μ is the vector of means, *Z* is the random effects design matrix, *u* represents genotypic response considered as random effects, whereas ε is the residual vector. The methods were implemented in the BWGS package in R [44].

#### 4.4.2. LASSO and Elastic Net

Least Absolute Shrinkage and Selection Operator (LASSO) penalizes the regression method, shrinking more estimates than Ridge Regression. LASSO shifts the less informative variables towards zero, and in the final model only the most significant coefficients are kept. The objective function of the model is represented as:

$$\underset{\beta }{\min}\overset{N}{\underset{i=1}{\sum }}{\left(y_{i}-x_{i}\prime \beta \right)}^{2}+\lambda_{L}\left|\beta \right|$$

where x_i_ is the marker genotype of landrace *i*, β is the marker effects, λ_L_ is a regularization parameter, and ││ is the L_1_ norm. The L_1_ penalty |β| regresses the effects towards zero more than the L_2_ penalty ${\left||\beta |\right|}^{2}$.

Elastic Net (EN) is the combination of both RR and LASSO regression [47]. The EN model shifts some of the variables towards zero and others are set to exactly zero, as in RR and LASSO. EN uses both the L_1_ and L_2_ penalties. The objective function of the model is represented as:

$$\underset{\beta }{\min}\overset{N}{\underset{i=1}{\sum }}{\left(y_{i}-x_{i}\prime \beta \right)}^{2}+\lambda_{E}\left(\left(1-\alpha \right)/2{\left||\beta |\right|}^{2}+\alpha \left|\beta \right|\right)$$

where λ_E_ is a regularization parameter.

#### 4.4.3. Bayesian Models

The Bayesian prediction models take into account prior marker effect distributions and are of the formula:

$$y=1_{n}\mu +X\beta +\varepsilon$$

where X represents the marker matrix incidence, and β is the vector of *k* marker effects. All the Bayesian models were implemented in the BWGS package in R [44].

##### Bayesian Ridge Regression

Bayesian Ridge Regression (BRR) is considered as the Bayesian version of RR-BLUP; the BRR shrinks the estimates of all the marker effects towards zero. The shrinkage is dependent on the sample size, but however independent of the effect size. The difference between BRR and RR-BLUP is between choosing the ridge parameter, in BRR a Gaussian prior that is independent and identically distributed (IID) with a common variance to all marker effects is used in the following form:

$$p\left(\beta_{R}|\sigma^{2}{}_{\beta_{R}}\right)=\overset{n}{\underset{j=1}{\prod }}N\left(\beta_{R_{j}}|0,\sigma^{2}{}_{\beta_{R}}\right)$$

here, $\beta_{R}$ is the vector of regression coefficient, and the marker effect prior variance is represented as $\sigma^{2}{}_{\beta_{R}}$. Then, the variance ($\sigma^{2}{}_{\beta_{R}}$) was assigned an inverse scaled χ^2^ density which includes a prior degree of freedom and scale as ${\mathrm{df}}_{\beta_{R}}$ and $S_{\beta_{R}}$ [35].

##### Bayes A

Bayes A (BA) uses a scaled-t prior distribution of marker effects, as all the markers can contribute differently towards the genetic variance. Therefore, it would not be correct to assume a common variance to all the markers, and a model with marker specific shrinkage can be more realistic [30]. BA shrinks the markers with effect closer to zero, but however does not strongly affect markers showing large effects [48]. A scaled-t prior density is assigned for shrinkage of markers. The BA model is implemented in two steps. In the first step the normal densities with zero mean and marker specific variance parameters are assigned to marker effects. In the second step, IID scaled-inverse χ^2^ densities are assigned to marker variances [49]. The prior densities for BA are denoted as follows:

$$p\left(\beta_{j},\sigma_{\beta j}^{2},S_{\beta }\right)=\left[\underset{k}{\prod }N\left(\beta_{jk}|0,\sigma_{\beta jk}^{2}\right)\chi^{-2}\left(\sigma_{\beta jk}^{2}{|\mathrm{df}}_{\beta },S_{\beta }\right)\right]\times G\left(\left(S_{\beta }\right)|r,s\right)$$

##### Bayes B

Bayes B (BB) uses the distribution prior where marker effects are assumed to be drawn from a scaled-*t* distribution. The marker effects are presumed as zero with probability, π, and they are drawn from a scaled-*t* distribution with probability 1-π. The difference between BA and BB is that the BB model assumes many markers having no effect at all, and thus π > 0 instead of π = 0 in case of BA [28]. This is considered as more realistic prior as most of the genomic region does not contain quantitative trait loci and therefore have zero effect [17]. The prior densities in BB used in the BWGS package as implemented in the BGL

$$p\left(\beta_{j},\sigma_{\beta }^{2},\pi \right)=\left\{\underset{k}{\prod }\left[\pi N\left(\beta_{jk}|0,\sigma_{\beta }^{2}\right)+\left(1-\pi \right)1\left(\beta_{jk}=0\right)\right]x^{-2}\left(\sigma_{\beta jk}^{2}{|\mathrm{df}}_{\beta },\;S_{\beta }\right)\right\}\;\times B\left(\pi |p_{0},\pi_{0}\right)\times G\left(S_{\beta }|r,s\right)$$

R package are represented as follows:

##### Bayes C

Bayes C (BC) is similar to BB except for a slab with Gaussian distribution instead of t-density in the case of BB [46]. BC model treats the probability of zero effect markers as unknown and estimates it instead of assigning it a predefined fixed as this could lead to affect the shrinkage of marker effects. BC was thus developed to address the shortcomings in BA and BB models [28]. BC is considered more flexible for the modeling of both oligogenic and polygenic traits [15,50]. The BC model implemented in BWGS uses the BGLR package library where BC is similar to the BB model except in that it estimates the variance parameter ($\sigma_{\beta }^{2}$) from the data, $p\left(\sigma_{\beta }^{2}\right)=x^{-2}\left(\sigma_{\beta }^{2}{|\mathrm{df}}_{\beta },S_{\beta }\right).$

#### 4.4.4. Reproducing Kernel Hilbert Spaces (RKHS)

RKHS is a semiparametric regression method which accounts for nonadditive effects. It is based on a kernel function and genetic distance among loci to regulate marker effect distribution [51]. RKHS is of the same form as RR-BLUP and GBLUP where *g = k* and can be represented as follows:

y=1μ+Kα+ε

where *y* and ε  are the same as previously described in GBLUP, whereas α is the random effects vector. As implemented in the BWGS package using the BGLR library the additive genetic effects $u\;\sim \;N\left(0,K\sigma_{g}^{2}\right)$, where K is the Gaussian reproducing kernel, $K\left(x_{i},x_{j}\right)=exp\left\{-\left[\left(x_{i}-x_{j}\right)\prime \left(x_{i}-x_{j}\right)\right]/h\right\}$, and $\sigma_{g}^{2}$ is the additive genetic variance.

### 4.5. Simulation Analysis

To obtain further insights regarding the predictive abilities of the GP models, two simulations were run based on modifying the number of landraces and by randomly selecting subsets of markers to optimize the number of markers required for the highest predictive ability. However, only the GBLUP model was used in the simulation analyses for comparison with normal and simulated runs. Pearson correlation between the phenotypic values and the GEBV was used to determine and compare the predictive ability of the models. The predictive ability of the models was assessed using 5-fold cross-validation. The whole set of landraces was divided randomly into five subgroups and four of them were used to estimate the GEBV’s of the fifth subset of landraces.

## Figures and Tables

**Table 1:** Phenotypic percentage distribution for yellow (YR), leaf (LR), and stem (SR) rust, in 2016 and 2017, under field conditions.

Disease-Year	Rust Infection Types				Heritability
	R (%)	MR (%)	MS (%)	S (%)	
**YR-2016**	12.4	19.5	22.0	46.0	0.97
YR-2017	13.0	21.9	18.2	46.9	0.97
LR-2016	21.5	7.2	13.1	58.0	0.98
LR-2017	9.0	11.4	8.1	71.2	0.97
SR-2017	10.1	13.5	14.7	61.5	0.97

**Table 2:** Pearson correlations at significance level alpha = 0.05 (*) between yellow (YR), leaf (LR) and stem (SR) rust trials, in 2016 and 2017, under field conditions.

	YR-2016	YR-2017	LR-2016	LR-2017	SR-2017
**YR-2016**		0.70*	−0.22	−0.26 *	−0.09
**YR-2017**	0.70 *		−0.26 *	−0.11	−0.14
**LR-2016**	−0.22	−0.26*		0.41 *	0.48 *
**LR-2017**	−0.26 *	−0.11	0.41 *		0.51 *
**SR-2017**	−0.09	−0.14	0.48 *	0.51 *	

**Table 3:** Predictive abilities of the models for yellow (YR), leaf (LR), and stem (SR) rust adult plant resistance under field conditions using nine different methods in wheat landraces from Afghanistan preserved in ICARDA’s gene bank.

Model	Disease-Year of Field Experiments				
	YR-2016	YR-2017	LR-2016	LR-2017	SR-2017
**GBLUP**	0.32 ± 0.02	0.30 ± 0.01	0.38 ± 0.01	−0.003 ± 0.05	0.30 ± 0.01
**RR**	0.32 ± 0.01	0.30 ± 0.01	0.38 ± 0.01	0.03 ± 0.04	0.302 ± 0.02
**LASSO**	0.31 ± 0.03	0.26 ± 0.02	0.36 ± 0.02	−0.03 ± 0.05	0.33 ± 0.02
**EN**	0.31 ± 0.02	0.28 ± 0.02	0.36 ± 0.02	−0.04 ± 0.05	0.33 ± 0.02
**BRR**	0.32 ± 0.02	0.30 ± 0.01	0.38 ± 0.01	0.04 ± 0.04	0.31 ± 0.02
**BA**	0.32 ± 0.02	0.30 ± 0.01	0.37 ± 0.01	0.09 ± 0.04	0.30 ± 0.02
**BB**	0.32 ± 0.02	0.30 ± 0.01	0.38 ± 0.01	0.04 ± 0.04	0.31 ± 0.02
**BC**	0.32 ± 0.01	0.30 ± 0.02	0.37 ± 0.01	0.04 ± 0.03	0.30 ± 0.02
**RKHS**	0.33 ± 0.01	0.29 ± 0.01	0.37 ± 0.01	0.05 ± 0.03	0.29 ± 0.01

**Table 4:** Spearman rank correlation coefficients between GEBVs for all genomic prediction methods.

	GBLUP	RR	LASSO	EN	BRR	BA	BB	BC	RKHS
**GBLUP**		0.97	0.82	0.82	0.97	1	0.97	1	1
**RR**	0.97		0.9	0.9	1	0.97	1	0.97	0.97
**LASSO**	0.82	0.9		1	0.9	0.82	0.9	0.82	0.82
**EN**	0.82	0.9	1		0.9	0.82	0.9	0.82	0.82
**BRR**	0.97	1	0.9	0.9		0.97	1	0.97	0.97
**BA**	1	0.97	0.82	0.82	0.97		0.97	1	1
**BB**	0.97	1	0.9	0.9	1	0.97		0.97	0.97
**BC**	1	0.97	0.82	0.82	0.97	1	0.97		1
**RKHS**	1	0.97	0.82	0.82	0.97	1	0.97	1	

## Data Availability

The data is already provided as Supplementary File S1, as advised by the reviewers.

## Supplementary Materials

The following are available online at https://www.mdpi.com/2223-7747/10/3/558/s1, Figure S1. Heatmap of marker relationship matrix of 363 wheat landraces showing relatedness (kinship) between the landraces. Supplementary File S1. The Genotypic data of 363 bread wheat landraces used in the study. Table S1. Genomic distribution of 11,428 SNPs markers on Wheat genome. Table S2. The 363 bread wheat landraces used in the study with original SeedID and origin information.
